# TNF-α inhibitor ameliorates immune-related arthritis and pneumonitis in humanized mice

**DOI:** 10.3389/fimmu.2022.955812

**Published:** 2022-08-09

**Authors:** Jian Gao, Jinlin Miao, Haoyang Sun, Xianghui Fu, Peiyan Zhang, Zhinan Chen, Ping Zhu

**Affiliations:** ^1^ Department of Clinical Immunology, National Translational Science Center for Molecular Medicine & Department of Cell Biology, PLA Specialized Research Institute of Rheumatoid & Immunology, Xijing Hospital, The Fourth Military Medical University, Xi’an, China; ^2^ National Center for International Research of Bio-targeting Theranostics, Guangxi Key Laboratory of Bio-targeting Theranostics, Collaborative Innovation Center for Targeting Tumor Diagnosis and Therapy, Guangxi Talent Highland of Bio-targeting Theranostics, Guangxi Medical University, Nanning, China

**Keywords:** TNF-α, immune checkpoint, immunotherapy, humanized mouse, pneumonitis, arthritis

## Abstract

**Objectives:**

This study aimed at establishing a mouse model of immune-related adverse in humanized BALB/c-hPD1/hCTLA4 mice to investigate their potential pathogenesis and explore therapeutic targets for immune-related arthritis and pneumonitis.

**Methods:**

Humanized BALB/c-hPD1/hCTLA4 mice were injected with vehicle or collagen-specific antibodies (CA) and immune checkpoint inhibitors (ICI, ipilimumab, anti-human CTLA-4; and nivolumab, anti-human PD-1), and some mice were treated with anti-TNF-α antibody, leading to the control, collagen antibody-induced arthritis (CAIA), CAIA+ICI and treatment groups. The severity of clinical arthritis and pneumonitis in mice was monitored longitudinally and the pathological changes in the joints and lungs were histologically analyzed and the contents of lung hydroxyproline were measured. The frequency of different subsets of T cells was analyzed by flow cytometry and multiplex immunofluorescency.

**Results:**

Compared with the control, the ICI group of mice developed the delayed onset of moderate degrees of arthritis while the CAIA+ICI group of mice exhibited the early onset of severe arthritis. Treatment with ICI caused severe pneumonitis, especially in the mice with CA. Flow cytometry analysis indicated a significantly higher frequency of splenic TNF-α^+^CD4^+^ and TNF-α^+^CD8^+^ T cells, but not other subsets of T cells tested, in the CAIA+ICI group of mice, relative to that in other groups of mice. Treatment with anti-TNF-α significantly mitigated the severity of arthritis and pneumonitis as well as deposition of collagen in lung of mice. The treatment also decreased the frequency of TNF-α^+^CD4^+^ and TNF-α^+^CD8^+^ T cells as well as effector memory T cells in the periphery lymph orangs and lungs of mice.

**Conclusions:**

We successfully established a humanized mouse model of ICI-related severe arthritis and pneumonitis with a higher frequency of TNF-α^+^ T cells, which were significantly mitigated by anti-TNF-α treatment. Conceptually, ICI treatment can induce multiple autoimmune-like diseases in autoimmune-prone individuals and TNF-α^+^ T cells may be therapeutic targets for intervention of immune-related arthritis and pneumonitis.

## Introduction

Immune checkpoints are a class of molecules that are widely expressed on the surface of activated and effector T cells. These molecules function to regulate the immune system and maintain immune homeostasis (1). Among them, CTLA-4 and PD-1 are the most studied molecules. The balance between their costimulatory and co-suppressive signals determines T cell activity. These immune checkpoints are crucial for maintaining peripheral T cell tolerance and protecting organs from autoimmune destruction, and serve as targets for tumor therapy (2). Immunotherapy with immune-checkpoint inhibitors (ICI), such as ipilimumab, pembrolizumab and nivolumab, has demonstrated to benefit patients with hematological malignancy and those with some types of solid malignant tumors (3–5).

Treatment with ICI usually induces strong T cell immune responses, but can lead to significant toxicities, immune-related adverse events (irAEs) in some recipients. The irAEs usually affect the lung, intestine, skin, pancreatic islets, joints, liver and other organs and are clinically similar to autoimmune diseases, such as pneumonitis, arthritis, colitis, hypothyroidism, liver dysfunction, vitiligo, type 1 diabetes and others (6–13). Previous studies have shown that treatment with both nivolumab and ipilimumab results in a higher adverse rate (14), particularly for the onset of arthritis and pneumonitis (15–18). Furthermore, patients with a history of autoimmune disease, such as rheumatoid arthritis, psoriasis, colitis or another one, are prone to the development of irAEs (19) and those patients are susceptible to a new autoimmune disease following ICI therapy (19–21).

Currently, the precise mechanisms underlying the pathogenesis of irAEs, especially for arthritis and pneumonitis, are incompletely understood. It is well known that the imbalance of pro-inflammatory and anti-inflammatory immune responses contributes to the pathogenesis of irAEs. For example, many pro-inflammatory effectors and memory T cells infiltrate the targeted tissues and organs (22–24). Recently, some investigations have reported that high levels of pro-inflammatory IL-6, IL-17 and GM-CSF secretion may contribute to the onset of irAEs (25–27). However, what types of effector T cells are and how they mediate the onset of irAEs have not been clarified, particularly for the ICI-related arthritis and pneumonitis in humans. In addition, there is no reliable animal model that mimics irAEs in humans and its clinical and pathological manifestations are different from those of conventional rheumatoid arthritis and pneumonitis. Therefore, it is urgently needed to establish animal models and explore the pathogenesis of ICI-related arthritis and pneumonitis to discover new therapeutic targets for intervention of the ICI-related arthritis and pneumonitis.

In this study, we attempted to establish a mouse model of ICI-related arthritis and pneumonitis in humanized BABL/c-hPD-1/hCTLA4 transgenic mice and characterized their pathogenesis as well as tested the therapeutic effect of anti-TNF-α on ICI-related arthritis and pneumonitis in mice. Our data indicated that treatment with ICI successfully induced severe arthritis and pneumonitis in mice, accompanied by a higher frequency of TNF-α^+^ T cells, particularly in those with collagen-specific antibodies. Treatment with anti-TNF-α significantly mitigated the severity of ICI-related arthritis and pneumonitis by minimizing TNF-α^+^ T cells in mice. Our findings suggest that TNF-α^+^ T cells may be crucial for the pathogenesis of ICI-related arthritis and pneumonitis, and therapeutic targets for intervention of these irAEs.

## Material and methods

### Animals

**Figure 1:**
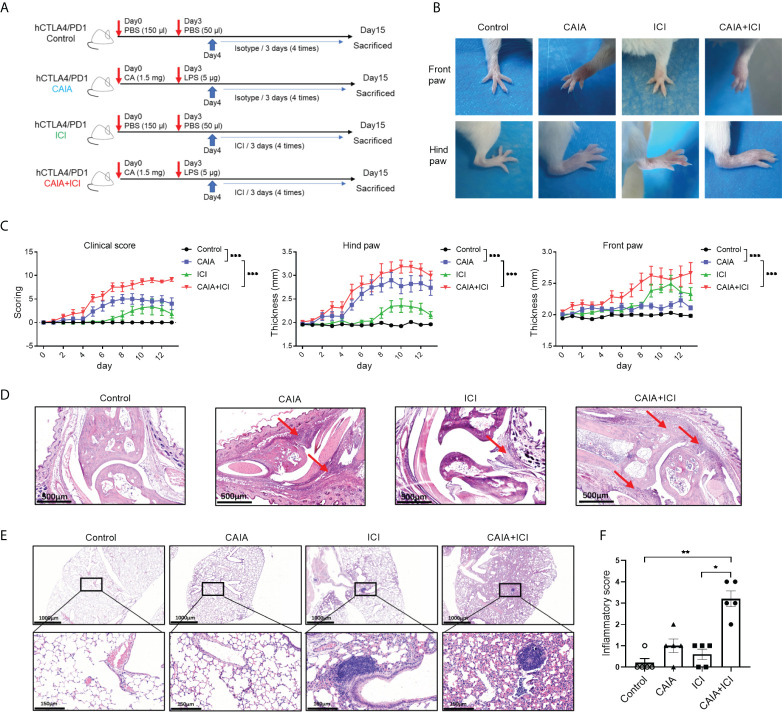
Establishment and histological characterization of ICI-related arthritis and pneumonitis in humanized mice. **(A)** A diagram illustrated the experimental design. **(B)** Representative images of paw swelling and ankle joints in mice. **(C)** The dynamics of clinical arthritis scores and paw thicknesses in mice. **(D)** Representative images of the ankle joints after hematoxylin-eosin (HE) staining in mice. Arrows indicate lymphocyte infiltrates and soft tissue injury. **(E)** Representative images of lung tissues in mice after HE staining. **(F)** inflammatory scores in lung tissues of mice. Data are representative images or presented as means ± SEM (n=6) of each group of mice. Statistical significance was analyzed by two-way ANOVA and *post hoc* Bonferroni test or the Kruskal-Wallis H test. ^*^ *p* < 0.05, ^**^ *p* < 0.01. ^***^ *p* < 0.001.

Female humanized BALB/c-hPD1/hCTLA4 mice (5-8 weeks of age, 17–22 g) were purchased from the Gempharmatech (Wilmington, USA), and were housed in a specific pathogen-free facility in the Animal Center of Air Force Medical University with a 12 h light-dark cycle. The animal experimental protocols were approved by the Institutional Animal Care and Use Committee of Air Force Medical University. The mice were randomized and injected intraperitoneally with vehicle PBS or the collagen-specific antibodies (1.5 mg/mouse, Chondrex, Redmond, Washington, USA) on day 0, and vehicle PBS or lipopolysaccharides (LPS, 5 µg/mouse) on day 3, followed by intraperitoneally injecting with control human IgG-Fc isotype control (200 µg/mouse, Athens, GA, USA) or ICI (ipilimumab and nivolumab, each 100 µg/mouse, TargetMol, Boston, USA) beginning on day 4 every three days for four times, leading to the control, CAIA, ICI and CAIA+ICI groups (**Figure 1A**). To test the therapeutic effect of anti-TNF-α, the mice were randomized and injected intraperitoneally with PBS throughout all injection time points as the control or collagen-specific antibodies (1.5 mg/mouse) on day 0 and LPS (5 µg/mouse) on day 3, followed by intraperitoneal treatment with ICI (100 µg/mouse) combined with InVivoMAb rat IgG1 isotype control (100 µg/mouse, BioXcell, USA) as the CAIA+ICI or InVivoMab anti-mouse TNF-α (100 µg/mouse, BioXcell, USA) every three days beginning on day 4 for 6 times as the treatment group (**Figure 3A**).

### Mouse arthritis scoring

The thicknesses of both front and hind paws were measured using a digital caliper in a blinded manner. The redness and swelling in interphalangeal, metacarpophalangeal, carpal and tarsal joints were scored on a scale of 0 to 4 (28). Score 0: Normal; 1: One joint has redness and swelling; 2: Two joints have redness and swelling; 3: Two joints have redness and swelling; and 4: Maximal redness and swelling of the entire paw without clearly anatomical definition.

### Histology

The animals were euthanized at the indicated times, and their left lungs were dissected and fixed in 4% paraformaldehyde. Their joints were fixed in 10% EDTA decalcification solution containing 4% neutral phosphate-buffered formalin that was exchanged every 5 days for 2 months. The left lung and joint samples were dehydrated and embedded in paraffin. The joint tissue sections (2 μm) were stained with hematoxylin and eosin (HE), and the lung tissue sections were stained with HE, Masson’s Trichrome and were subjected to immunohistochemistry (IHC) analysis.

### Histological scoring

The HE-stained images were scored for inflammation (29) as score 0: no inflammation; 1: a few inflammatory infiltrates; 2: mild degrees of inflammation; 3: moderate degrees of inflammation; and 4: severe levels of inflammation. The Masson’s Trichrome-stained images were scored for fibrosis using the Ashcroft Fibrosis Score system (30) as grade 0: normal lung; 1: slight fibrous thickening of the bronchiole wall or alveolar wall; 3: no obvious damage in the alveolar structure, but a more fibrous thickening of the bronchiole wall or alveolar wall; 5: Obvious destruction of alveolar structures with fibrous strips and small fibrous foci. 7: Severe destruction of alveolar structures with large areas of honeycomb changes; 8: almost complete pulmonary fibrosis (levels 2, 4, and 6 are the transitional stages of the aforementioned levels).

### Multiplex immunofluorescency assay

Multiplex immunofluorescent analyses were performed as previously described (31). Briefly, the tissue sections were rehydrated and subjected to antigen retrieval in TRIS-EDTA (pH = 9) buffer. The sections were permeabilized with 0.5% Triton X100, blocked with 5% goat serum in PBS and sequentially incubated with anti-CD4, anti-CD8 (Abcam, USA) and anti-TNF-α (Cloud-Clone, China). After being washed, the bound antibodies were reacted with goat anti-rabbit IgG (Panovue, Beijing, China) and stained using the TSA indirect kit (PerkinElmer), according to the manufacturer’s instructions. The sections were microwaved to remove the primary and secondary antibodies, washed, and blocked again using blocking solution. The sections were stained with second primary antibody, and repeated with the process, as described above. Finally, the sections were stained with DAPI. The fluorescent signals were photoimaged and the images were analyzed using Inform software (version 3.0).

### Hydroxyproline

The contents of hydroxyproline in the right lung tissues were measured for the degrees of pulmonary fibrosis using the hydroxyproline assay kit (Nanjing Jiancheng Bioengineering Institute, Jiangsu, China), according to the manufacturer’s protocol. Briefly, about 100 mg of lung tissues from each mouse were homogenized in 1 ml of lysis buffer and heated in an oven at 95°C for 20 min. After adjusting the pH to 6.0-6.8, the lysate volumes were expanded to 10 ml. Each 3-4 ml of lysates was mixed with 20-30 mg activated carbon and centrifuged at 1500 g for 10 min. The supernatants (1 ml each) were reacted with the reagents of 1, 2 and 3 at 60° C for 20 min. The absorbance of each sample was measured at 550 nm to determine the amount of hydroxyproline. The contents of hydroxyproline in the lung tissues were calculated as µg hydroxyproline per mg wet lung weight, using the established standard curve.

### Flow cytometry analysis

Splenic, lymph node and peripheral blood mononuclear cells were prepared from individual mice and stained with PE/Dazzle™ 594 anti-mouse CD3, FITC anti-mouse CD4, APC-Fire750 anti-mouse CD8a, PE/Cyanine7 anti-mouse/human CD44, APC anti-mouse CD62L (BioLegend, San Diego, USA) on ice. Some cells were treated with cell activation cocktail in the presence of brefeldin A (BioLegend, Cat: 423303) at 37°C for 6 h. The cells were stained with fluorescent antibodies against surface markers, fixed and permeabilized using an intracellular staining kit (BioLegend, Cat: 421002), followed by intracellularly staining with PE/Cyanine7 anti-mouse TNF-α, PerCP/Cyanine5.5 anti-mouse IFN-γ, PE anti-mouse IL-4, APC anti-mouse IL-17A (BioLegend). Other cells were stained with antibodies against surface markers, fixed and permeabilized using the Foxp3/Transcription Factor Staining Buffer (eBioscience, Waltham, MA, USA), followed by intracellularly staining with PE-anti-Foxp3 (eBioscience). The cells were analyzed by flow cytometry using FlowJo software 10.6 (Tree Star, Ashland, OR).

### Statistical analysis

Statistical analyses were performed using GraphPad Prism software (version 8.3) or SPSS (version 23). All data are presented as the mean ± SEM. For normalized data, the difference among the groups was analyzed by one-way ANOVA and *post hoc* Bonferroni or Tamhane’s T2 analysis. The difference between the groups was analyzed by a two-tailed Student’s *t* test. For skewed data, the difference among groups was analyzed by the Mann-Whitney *U* test or Kruskal-Wallis *H* test. The significance was considered statistically significant when a *p*-value of < 0.05 (^*^ *p* < 0.05, ^**^ *p* < 0.01, ^***^ *p* < 0.001).

## Results

### Treatment with ICI induces severe arthritis and pneumonitis in collagen antibody-injected humanized BALB/c-hPD1/hCTLA4 mice

To establish a mouse model of immune-related arthritis and pneumonitis, humanized BALB/c-hPD1/hCTLA4 mice were randomized and injected intraperitoneally with PBS or collagen-specific autoantibodies and LPS, followed by challenging multiple times with vehicle or ICI, leading to the control, CAIA, ICI and CAIA+ICI groups (**Figure 1A**). The thicknesses of paws and the severity of clinical arthritis were scored longitudinally. Compared with the controls, there was an obvious increase in paw edema in the CAIA and ICI groups of mice (**Figure 1B**). The clinical arthritis scores and the thicknesses of hind paws significantly increased at 8-11 days post-challenge in the ICI group of mice, indicating that ICI treatment alone induced clinical arthritis (**Figure 1C**). In contrast, early onset of severe arthritis was detected in both the CAIA and CAIA+ICI groups of mice, and the clinical arthritis scores and the thicknesses of hind paws in the CAIA+ICI group were more severer than that in the CAIA group of mice (*p* < 0.001, **Figure 1C**). A similar pattern of front paw thicknesses was observed in the different groups of mice, except that the mice in the CAIA group displayed low degrees of frond paw thicknesses in our experimental condition.

Histological analyses exhibited that compared with the control mice, there was a significantly obliterated joint space with some inflammatory infiltrates in soft tissues in the CAIA and ICI groups of mice. In contrast, many inflammatory cells infiltrates occupied the completely obliterated joint space with high degrees of soft tissue inflammation in the CAIA+ICI group of mice (**Figure 1D**). While there was not much change in the lung tissues with a few inflammatory cells infiltrates in the alveolar of the CAIA and ICI groups of mice, more alveolar damage and severer inflammation were observed in the lung tissues from the CAIA+ICI group of mice (**Figure 1E**). Quantitative analysis revealed that there was a significant difference in inflammatory scores among the groups of mice and the inflammatory scores in the CAIA+ICI group were significantly greater than that of the ICI group of mice (*p* < 0.05, **Figure 1F**). Collectively, these data indicated that treatment with ICI induced severe arthritis and pneumonitis in collagen antibody-injected humanized BALB/c-hPD1/hCTLA4 mice.

### Higher frequency of TNF-α secreted T cells exists in the CAIA+ICI groups of mice

**Figure 2:**
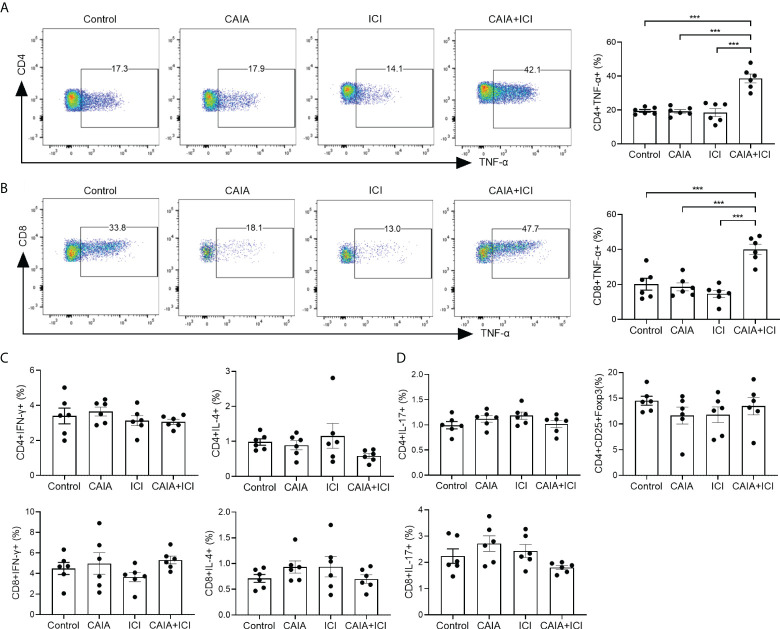
Flow cytometry analyses of different subsets of functional splenic T cells in mice. Splenic mononuclear cells were isolated from individual groups of mice and stained with the indicated fluorescent antibodies. Some cells were fixed, permeabilized and intracellularly stained with the indicated antibodies. The frequency of different sunsets of splenic functional T cells was characterized by flow cytometry. The cells were gated on living cells and then on CD4^+^ or CD8^+^ T cells. The data were analyzed Flowjo software. **(A, B)** Representative flow charts (left) and quantitative results (right) of splenic TNF-α secreting CD4^+^ or CD8^+^ T cells in mice. **(C, D)** Quantitative analysis of the frequency of the indicated functional splenic T cells. Data are representative flow charts or presented as means ± SEM (n=6) of each group of mice from two separate experiments. Statistical significance was tested by one-way ANOVA and *post hoc* Bonferroni test. ^***^ *p* < 0.001.

To understand the potential role of pro-inflammatory T cells, the percentages of splenic cytokine-secreted CD4^+^ and CD8^+^ T cells in individual mice were analyzed by flow cytometry. There was no significant difference in the frequency of splenic TNF-α^+^CD4^+^ or TNF-α^+^CD8^+^ T cells among the control, CAIA and ICI groups of mice while the frequency of splenic TNF-α^+^CD4^+^ or TNF-α^+^CD8^+^ T cells in the CAIA+ICI group was significantly higher than that of other groups of mice (*p <*0.001, **Figures 2A, B**). Further analyses exhibited that there was no significant difference in the frequency of splenic IFN-γ^+^, IL-4^+^, IL-17^+^ T cells and CD4^+^CD25^+^Foxp3^+^ Tregs among these groups of mice (**Figures 2C, D**). Hence, high frequency of TNF-α^+^CD4^+^ and TNF-α^+^CD8^+^ T cells existed, probably contributing to the pathogenesis of severe arthritis and pneumonitis in the collagen antibody-injected and ICI-treated humanized BALB/c-hPD1/hCTLA4 mice.

### Treatment with anti-TNF-α significantly mitigates the severity of arthritis and pneumonitis in the collagen antibody-injected and ICI-treated humanized BALB/c-hPD1/hCTLA4 mice

**Figure 3:**
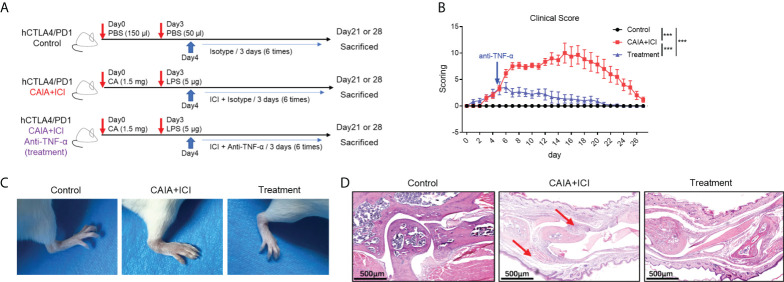
Anti-TNF-α treatment ameliorates the severity of ICI-related arthritis and pneumonitis in humanized mice. **(A)** A diagram illustrated the experimental design. **(B)** The dynamics of clinical arthritic scores in mice. **(C)** Representative images of paw swelling and ankle joints in mice. **(D)** Representative images of the ankle joints in mice after HE staining. Arrows indicate lymphocyte infiltrates and soft tissue injury in mice. Data are representative images or presented as means ± SEM of each group (n=6) of mice. Statistical significance was analyzed by one-way ANOVA and host hoc Bonferroni test or Kruskal-Wallis *H* test. ^***^ *p* < 0.001.

**Figure 4:**
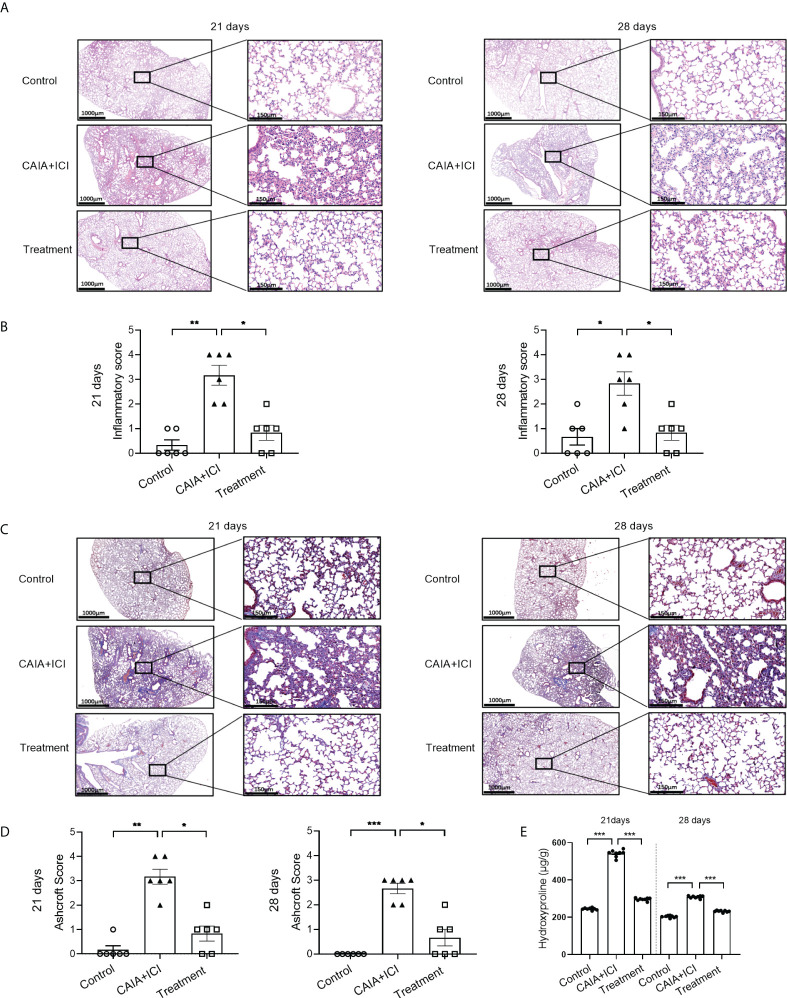
Anti-TNF-α treatment mitigates the degrees of lung inflammation in mice. **(A, C)** Representative images of lung tissue sections from mice on day 21 and 28 post induction after HE and Masson staining, respectively. **(B)** Lung inflammatory scores in mice. **(D)** Lung Ashcroft scores in mice. **(E)** The contents of lung hydroxyprolines in mice on day 21 and 28 post induction. Data are representative images or presented as means ± SEM of each group (n=6) of mice. Statistical significance was calculated by the Kruskal-Wallis H test ^*^ *p* < 0.05, ^**^ *p* < 0.01, ^***^ *p* < 0.001.

To determine the importance of TNF-α in the pathogenesis of severe arthritis and pneumonitis, humanized BALB/c-hPD1/hCTLA4 mice were randomized and injected intraperitoneally with vehicle as to the control or collagen-specific antibody and LPS, followed by injecting with ICI as the CAIA+ICI (**Figure 3A**). Some mice in the CAIA+ICI group were treated with anti-TNF-α and served as the treatment group. Longitudinal observations revealed that the mice in the CAIA+ICI group gradually developed severe arthritis post induction, with the highest clinical arthritis score at 13 days, but the clinical arthritis scores rapidly decreased after treatment with anti-TNF-α, and gradually declined and disappeared at 21 days post induction (**Figure 3B**). Supportively, treatment with anti-TNF-α almost abrogated the paw swelling in mice (**Figure 3C**). Histological analyses indicated that obvious cartilage destruction, inflammatory articular tissues, and disoriented joint space were observed in the CAIA+ICI group of mice, which were remarkably improved as only minimal inflammation in the articular tissue and little disorientation in the joint space without evidence of cartilage destruction were detected in the treatment group of mice (**Figure 3D**). Histological analysis of the lung tissues revealed that a large number of inflammatory cells infiltrates the lung tissues, leading to atelectasis at 21 and 28 days post induction in the CAIA+ICI group of mice, which were significantly mitigated in the treatment group of mice (**Figure 4A**). The inflammatory cells were mainly lymphocytes with few macrophages. Quantitative analysis indicated that treatment with anti-TNF-α dramatically decreased inflammatory scores in the lung of mice, compared with the CAIA+ICI group (**Figure 4B**). Further Masson staining revealed high levels of collagen deposition in the lung, accompanied by obvious lung damages in the CAIA+ICI group of mice, which were remarkably ameliorated in the treatment group of mice (**Figure 4C**). Quantitative analysis revealed that the Ashcroft pulmonary fibrosis scores in the treatment group were significantly lower than that in the CAIA+ICI group of mice (**Figure 4D**). Furthermore, the contents of hydroxyproline in the lungs of the treatment group were also significantly reduced, relative to that in the CAIA+ICI group of mice (**Figure 4E**).

### Treatment with anti-TNF-α reduces the frequency of TNF-α^+^ T cells in the collagen antibody-injected and ICI-treated humanized BALB/c-hPD1/hCTLA4 mice

**Figure 5:**
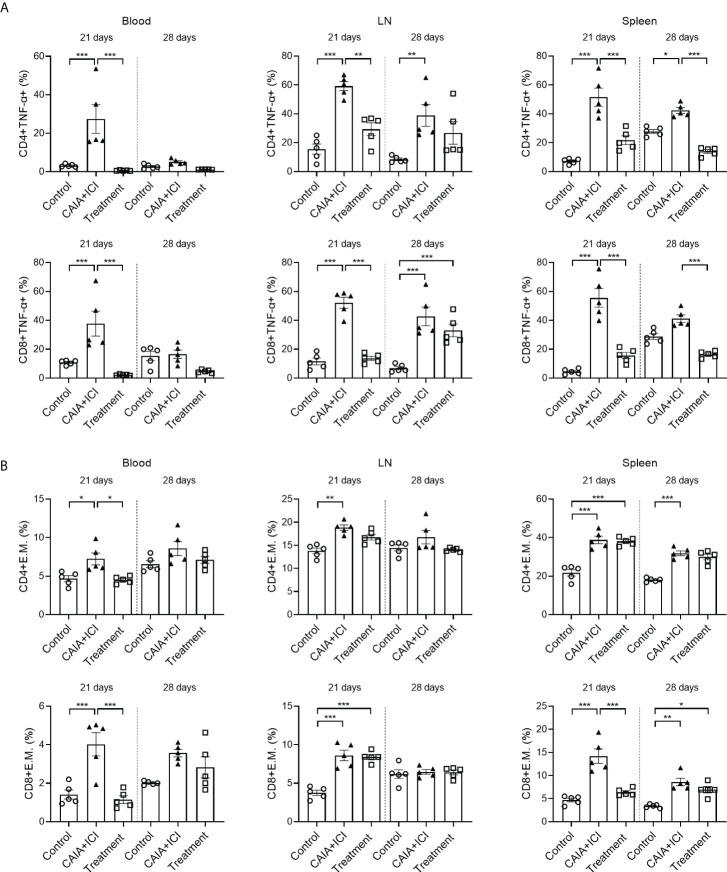
Anti-TNF-α treatment mitigates the frequency of TNF-α secreting T and effector memory T cells in mice. **(A)** Quantitative analyses of the frequency of TNF-α^+^CD4^+^ (top) and TNF-α^+^CD8^+^ (bottom) T cells in the peripheral blood, lymph nodes and spleens of individual mice. **(B)** Quantitative analyses of the frequency of CD44^+^CD62L^-^CD4^+^ (top) and CD44^+^CD62L^-^CD8^+^ (bottom) T cells in the peripheral blood, lymph nodes and spleens of individual mice. Data are presented as means ± SEM of each group (n=6) of mice. Statistical significance was analyzed by one-way ANOVA and *post hoc* Bonferroni test. ^*^ *p* < 0.05, ^**^ *p* < 0.01, ^***^ *p* < 0.001.

To understand the therapeutic action of anti-TNF-α, splenic, lymph node and peripheral blood mononuclear cells were isolated from individual mice and the frequency of TNF-α^+^CD4^+^ and TNF-α^+^CD8^+^ T cells were analyzed by flow cytometry. As shown in **Figure 5A**, in comparison with the control mice, the percentages of peripheral blood, lymph node and splenic TNF-α^+^CD4^+^ and TNF-α^+^CD8^+^ T cells in the CAIA+ICI group significantly increased, which were significantly mitigated or abrogated in the treatment group. Analysis of effector memory CD44^hi^CD62L^lo^ CD4^+^ and CD8^+^ T cells revealed that compared with the control mice, the percentages of peripheral blood, lymph node and splenic effector memory CD4^+^ and CD8^+^ T cells significantly increased, except for the frequency of peripheral blood and lymph node effector memory T cells at 28 days post induction (**Figure 5B**). In contrast, treatment with anti-TNF-α dramatically mitigated or abrogated the increase in the frequency of peripheral blood and splenic effector memory CD8^+^ T cells in the mice at 21 days post induction, but not at 28 days post induction (**Figure 5B**).

In addition, IFN-γ, IL-4 and IL-17 secreting T cells were also detected and analysed. Treatment with anti-TNF-α significantly mitigated the frequency of splenic IFN-γ^+^CD8^+^ T cells at 28 days, related to that in the CAIA+ICI group (**Supplementary Figures 1A**). Besides, treatment with anti-TNF-α increased splenic IL-4 producing T cells at 21days (**Supplementary Figures 1B**). And compared with the control, a significantly higher frequency of peripheral blood IL-17^+^CD4^+^ and IL-17^+^CD8^+^ T cells was detected in the CAIA+ICI group, which was significantly mitigated in the treatment group of mice. Moreover. the percentages of Tregs in the CAIA+ICI group significantly increased relative to that in the controls, but they were dramatically reduced in the treatment group at 21 days post induction (**Supplementary Figures 2B**).

**Figure 6:**
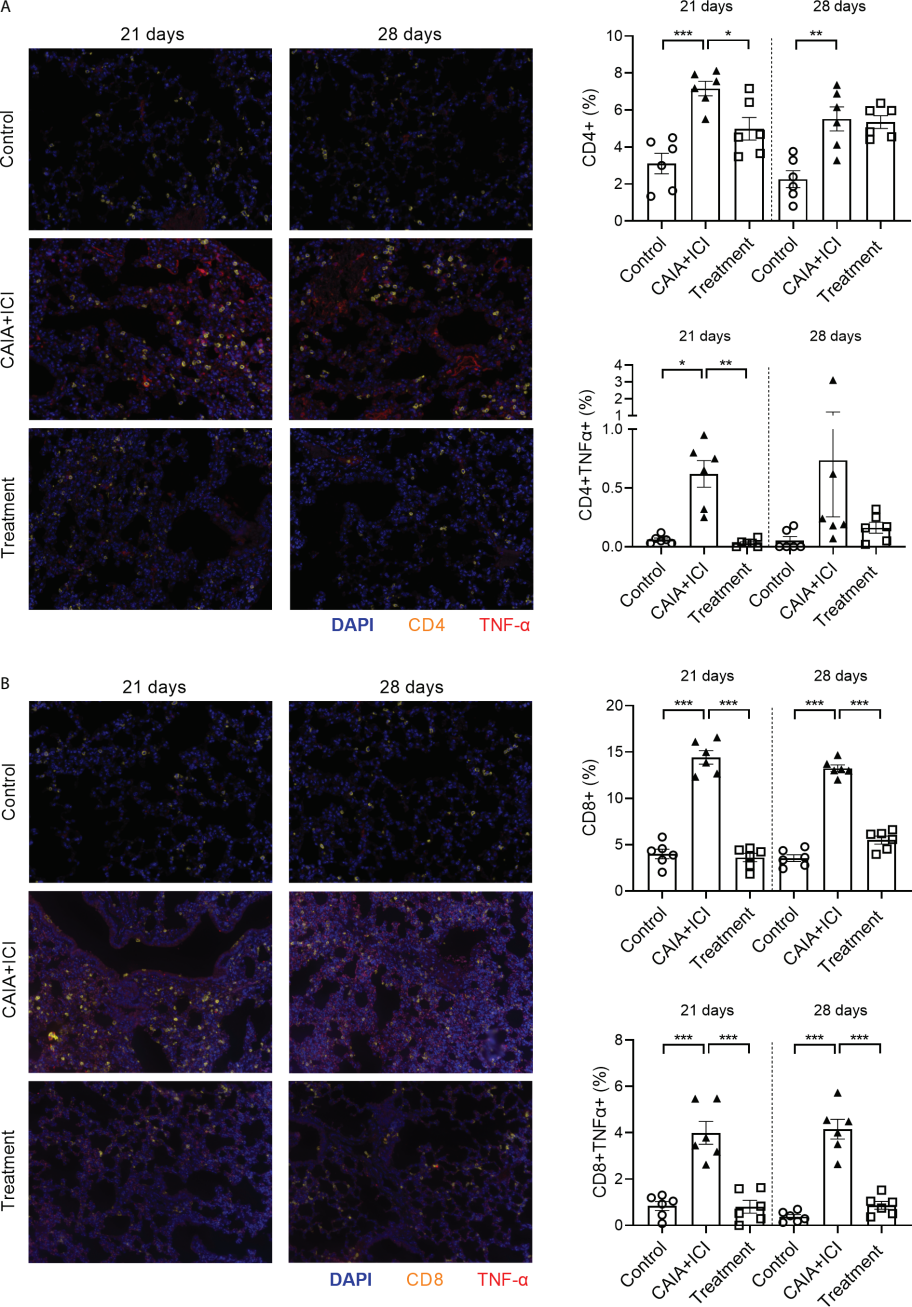
Anti-TNF-α treatment reduces TNF-α secreting T cell infiltrates in the lung of mice. The lung tissue sections from the indicated groups of mice on day 21 or 28 post induction were stained with fluorescent anti-mouse CD4 or anti-mouse CD8, anti-TNF-α and nuclear-stained with DAPI. The lung tissue sections were scanned by Vectra Polaris. **(A, B)** Representative fluorescent images of lung tissue sections and quantitative analysis of the frequency of CD4^+^, TNF-α^+^CD4^+^, CD8^+^ and TNF-α^+^CD8^+^ T cells in mice. Data are representative fluorescent images of lung tissue sections or presented as means ± SEM of each group (n=6) of mice. Statistical significance was analyzed by one-way ANOVA and *post hoc* Bonferroni test. ^*^ *p* < 0.05, ^**^ *p* < 0.01, ^***^ *p* < 0.001.

Multiplex immunofluorescency analyses revealed that compared with the controls, the percentages of CD4^+^, TNF-α^+^CD4^+^, CD8^+^ and TNF-α^+^CD8^+^ T cells in the lungs significantly increased in the CAIA+ICI group, which were dramatically mitigated or abrogated in the treatment group of mice, except that there was no therapeutic effect on the frequency of CD4^+^ T cells and there was no significant difference in the frequency of TNF-α^+^CD4^+^ T cells in the lungs among the different groups of mice on day 28 (**Figures 6A, B**). Collectively, treatment with anti-TNF-α significantly mitigated or abrogated the altered frequency of TNF-α^+^ T cells in the periphery and target lungs of mice.

## Discussion

Since ICI are used in the clinic the irAEs have been reported in the recipients and affect their life quality. Currently, there is no reliable animal model available because wild-type mice are tolerant to high doses of anti-mouse CTLA-4 and PD-1 antibodies (23). In addition, because the irAEs may be drug-specific, it is difficult to model the irAEs of anti-human CTLA-4 or PD-1 with anti-mouse ICI. Besides, the cross-species binding affinities of murine PD-1 to human PD-L1 and of human PD-1 to murine PD-L1 are similar to the affinities of same-species binding (32). Similarly, CTLA-4 can binds its B7-1 and B7-2 ligands by a conserved hydrophobic sequence (33). Consequently, hCTLA-4 or hPD-1 is likely to bind to mouse B7 or PD-L1 in humanized mice, and perform their biological function (32, 33). Accordingly, we explored establishing a mouse model of irAEs using humanized BALB/c-hPD1/hCTLA4 mice. In clinical patients, although irAE such as arthritis and pneumonitis may occur with monotherapy, in animal model studies, anti-CTLA4 or anti-PD1 monotherapy cannot induce significant arthritis and pneumonitis, and the combination of the both is used to mimic the clinical manifestations (23). Therefore, co-administration was used for model construction. We found that injection with ICI induced severe arthritis and pneumonitis in the mice, severer diseases in the mice that had been injected with CA, accompanied by a higher frequency of TNF-α^+^ T cells in the mice. Pathological analyses revealed many inflammatory lymphocyte infiltrates, but few macrophages, in the lungs of mice. The results were different from bleomycin-induced lung injury, which has a massive accumulation of mast and foam cells in the lungs (29, 34). Therefore, we successfully established a new mouse model of ICI-related arthritis and pneumonitis in humanized BALB/c-hPD1/hCTLA4 mice. Conceptually, ICI treatment can induce multiple autoimmune-like diseases simultaneously.

Previous studies have shown that the change in the frequency of T cells, particularly for Tregs, is associated with the pathogenesis of irAEs (23, 35). Recent studies have noted that high levels of pro-inflammatory cytokines may contribute to the pathogenesis of irAEs (18, 26, 27). In this study, we found that combination of CAIA and ICI, but not their alone, caused significantly higher frequency of TNF-α secreting CD4^+^ and CD8^+^ T cells and higher percentages of effector memory T cells, but not IFN-γ, IL-4, and IL-17 secreting T cells and Tregs, in the circulation and peripheral lymph organs as well as in the lungs of humanized mice. It is well known that TNF-α through its receptor of TNFR1 promotes inflammation, and implicates in a wide range of autoimmunity (36, 37). More importantly, treatment with anti-TNF-α significantly mitigated the severity of ICI-related arthritis and pneumonitis, consistent with previous reports (38–40). Our findings extended previous observations that TNF-α inhibitor benefits patients with autoimmune diseases (41–44) and the therapeutic efficacy of TNF-α inhibitor is better than traditional corticosteroids for patients with immune-related colitis (45, 46). Mechanistically, we found that treatment with anti-TNF-α dramatically reduced the frequency of TNF-α secreting CD4^+^ and CD8^+^ T cells and reduced their infiltration in the lungs of the humanized mice. Moreover, the treatment also significantly mitigated the percentages of effector memory T cells in the humanized mice, similar to the previous studies (23, 47), suggesting that effector memory T cells may also participate in the pathogenesis of ICI-related arthritis and pneumonitis. To the best of our knowledge, these novel data indicate that TNF-α secreting T cells contribute to the pathogenesis of ICI-related arthritis and pneumonitis in the humanized mice. Therefore, our findings may shed light on the pathogenesis of ICI-related arthritis and pneumonitis, and TNF-α secreting T cells may be therapeutic targets for intervention of ICI-related arthritis and pneumonitis.

In this study, we found that many TNF-α secreting CD4^+^ and CD8^+^ T cells infiltrated into the lung lesions of the CAIA+ICI group of mice. These indicated that TNF-α secreting T cells promoted the inflammatory responses and exacerbated lung damage by accumulating in the alveolar septa. Alternatively, TNF-α^+^ T cells may indirectly promote the synthesis of hydroxyproline through secreting TNF-α, leading to the exudation and deposition of collagen fibrin in lung tissues (48, 49). These support the notion that TNF-α is crucial for pulmonary collagen deposition (50). Indeed, anti-TNF-α treatment, not only mitigated TNF-α-secreting CD4^+^ or CD8^+^ T cell infiltrates in the lung tissues, but also reduced pulmonary inflammation and improved the alveolar morphology in the humanized mice. As reported in the previous literatures, TNF-α has been shown to increase the activation of both CD4^+^ and CD8^+^ T cells, including augmenting levels of IL-2R, enhancing T cell proliferation, and increasing cytokine production such as TNF-α (51–53). Thus, anti-TNF-α may suppresses this effect and lead to the reduction of both CD4^+^ and CD8^+^ T cell percentages. What’s more, our findings showed that anti-TNF-α could only regulated TNF-α secreting in T cells, rather than the other cytokines (like IFN-γ, IL-4 and IL-17), but the specific mechanism remains to be further investigated. Our findings extended previous observations that TNF-α inhibitors can reduce serum TNF-α levels to ameliorate inflammation (54–56). We are interested in further investigating how TNF-α-secreting T cells contribute to the pathogenesis of ICI-related arthritis and pneumonitis.

In addition, previous studies have illustrated that TNF-α inhibitors did not affect the tumor rejection (57, 58). Recently, the clinical trials illustrated that patients with developed severe colitis continued the immune-checkpoint inhibitors therapy while co-administering Infliximab or certolizumab, and almost all the patients displayed reduced colitis symptoms whereas overall disease stability was observed (59, 60). In a mouse melanoma model, the author demonstrated that TNF impairs the accumulation of CD8^+^ T cells in tumor-draining lymph nodes and tumors in a TNFR1-dependent manner (61, 62). Another study extended the concept by showing the role played by TNF in promoting activation-induced cell death (AICD) of CD8^+^ tumor-infiltrating lymphocytes (TILs) upon anti-PD-1 and anti-CTLA-4 combination therapy in mice (57). Mechanistically, TNF blockade prevented anti-PD-1-induced AICD of TILs and decreased their PD-L1 and TIM-3 expression. The therapeutic efficacy of the combination in mouse cancer models demonstrated that the efficient control of inflammatory bowel disease symptoms by TNF blockers and even enhanced the tumor rejection (57). Consequently, emerging evidence and recent clinical trials suggest that TNF inhibitors are safe and beneficial in the treatment of patients with cancer and irAEs.

In summary, our study successfully established a mouse model of immune-related adverses that recapitulated partial manifestations of ICI-related arthritis and pneumonitis in humans. Furthermore, our data indicated that a high frequency of TNF-α-secreting T cells contributed to the pathogenesis of ICI-related arthritis and pneumonitis as anti-TNF-α treatment significantly ameliorated the severity of ICI-related arthritis and pneumonitis. These suggest that TNF-α-secreting T cells may be therapeutic targets for intervention of ICI-related arthritis and pneumonitis.

## Data availability statement

The raw data supporting the conclusions of this article will be made available by the authors, without undue reservation.

## Ethics statement

The animal study was reviewed and approved by National Translational Science Center for Molecular Medicine.

## Author contributions

PZ and ZC conceived the study and revised manuscript. JG and JM performed most of the experiments and statistical analysis, and wrote the manuscript. HS and PYZ participated in the experiments. XF contributed to instruct the technological process of the study. All authors contributed to the article and approved the submitted version.

## Funding

The research was supported by the National Natural Science Foundation of China (NO. 92169211) and the Natural Science Basis Research Plan in Shaanxi Province of China (NO. 2019ZY-CXPT-03-01).

## Conflict of interest

The authors declare that the research was conducted in the absence of any commercial or financial relationships that could be construed as a potential conflict of interest.

## Publisher’s note

All claims expressed in this article are solely those of the authors and do not necessarily represent those of their affiliated organizations, or those of the publisher, the editors and the reviewers. Any product that may be evaluated in this article, or claim that may be made by its manufacturer, is not guaranteed or endorsed by the publisher.
