# Supplementary material for: TNF-α inhibitor ameliorates immune-related arthritis and pneumonitis in humanized mice
Source: Front Immunol. 2022 Aug 9;13:955812. doi: 10.3389/fimmu.2022.955812 (PMC9396351; doi:10.3389/fimmu.2022.955812)
Supplement: Supplementary file 1 [file DataSheet_1.docx]

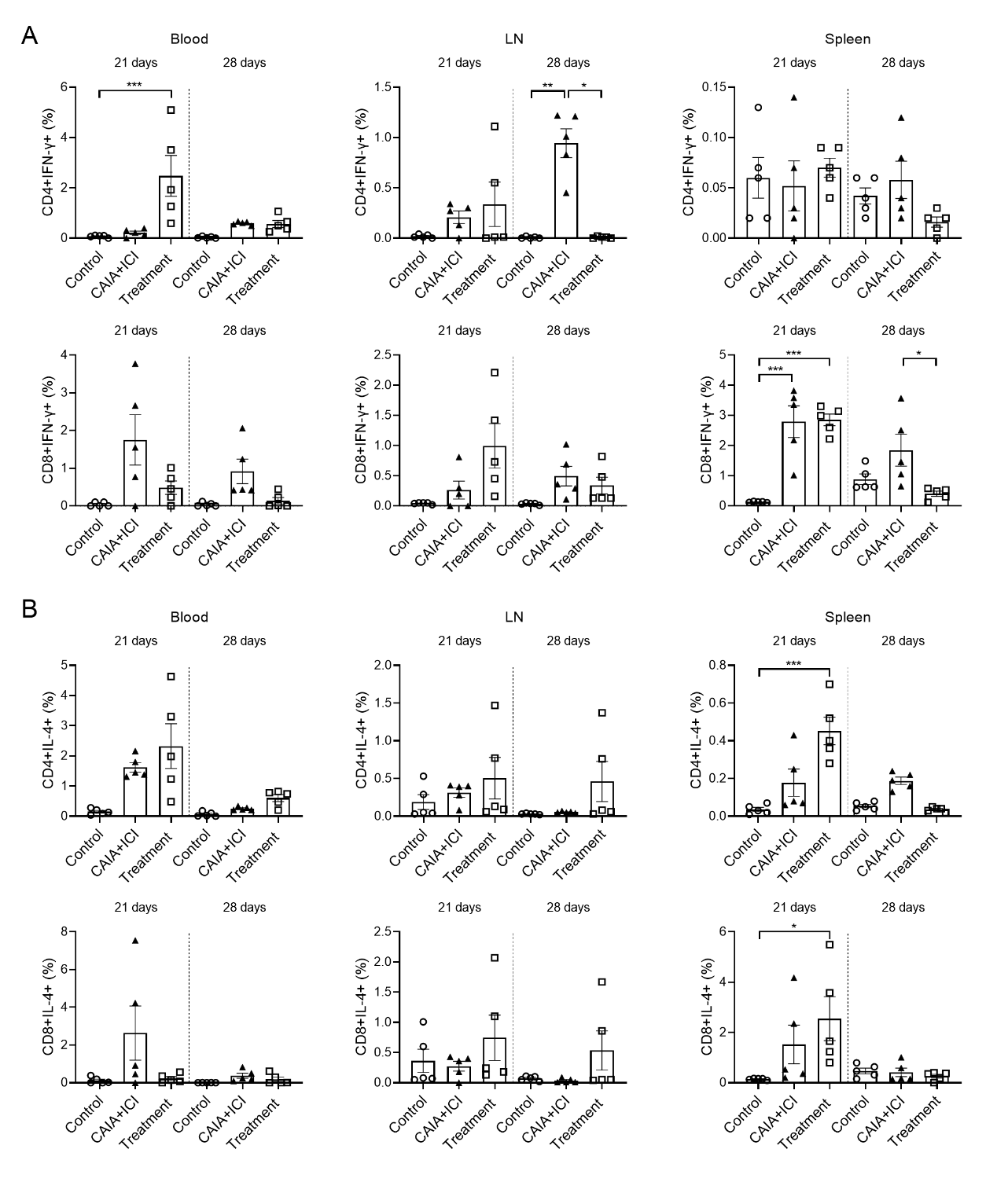


**Supplementary Figure 1. Quantitative analyses of the frequency of IFN-γ- and IL-4 secreting T cells in mice.** (A) Quantitative analyses of the frequency of IFN-γ^+^CD4^+^ (top) and IFN-γ^+^CD8^+^ (bottom) T cells in the peripheral blood, lymph nodes and spleen of mice. (B) Quantitative analyses of the frequency of IL-4^+^CD4^+^ (top) and IL-4^+^CD8^+^ (bottom) T cells in mice. Data are expressed as the means ± SEM of each group (n=5) of mice. Statistical significance was analyzed by one-way ANOVA and post hoc Bonferroni test. ^*^*p* < 0.05, ^**^*p* < 0.01, ^***^*p* < 0.001.


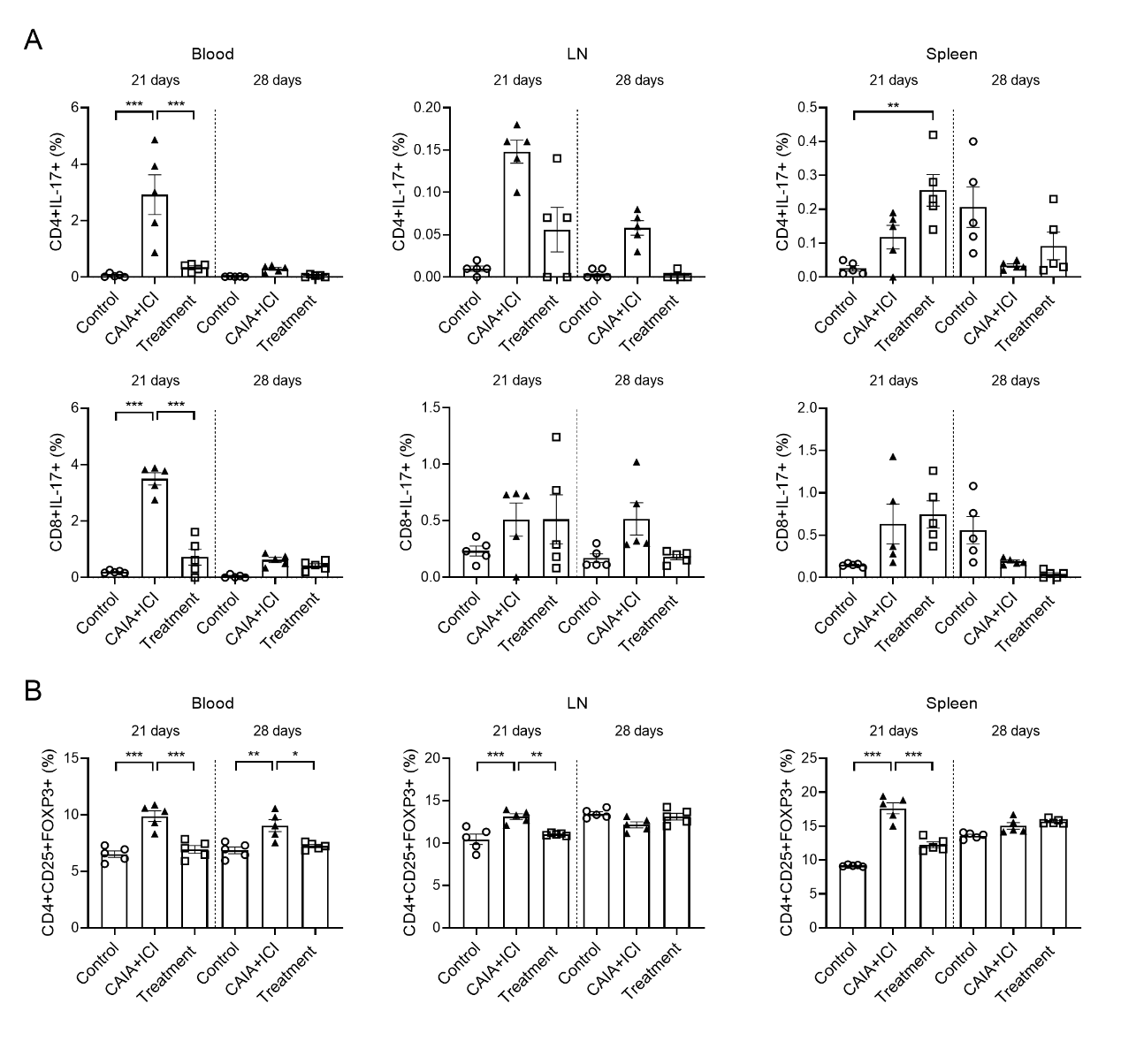


**Supplementary Figure 2. Quantitative analyses of the frequency of IL-17 secreting T cells and Tregs in mice.** (A) Quantitative analyses of the frequency of IL-17^+^CD4^+^ (top) and IL-17^+^CD8^+^ (bottom) T cells in the peripheral blood, lymph nodes and spleen of mice. (B) Quantitative analyses of the frequency of Tregs (CD4^+^CD25^+^Foxp3^+^) in mice. Data are presented as means ± SEM of each group (n=5) of mice. Statistical significance was analyzed by one-way ANOVA and post hoc Bonferroni test. ^*^*p* < 0.05, ^**^*p* < 0.01, ^***^*p* < 0.001.
